# Predictive value of longitudinal changes of serum matrix metalloproteinase-9 and brain-derived neurotrophic factor in acute ischemic stroke

**DOI:** 10.3389/fnagi.2022.952038

**Published:** 2022-08-25

**Authors:** Youjia Li, Xiaoyan Han, Songbao Luo, Huiqin Huang, Xinyan Huang, Miaochang Li, Yan Huang, Ying Chen, Zhenmei Wu

**Affiliations:** Department of Neurology, The First People’s Hospital of Zhaoqing, Zhaoqing, China

**Keywords:** neuroinflammation, matrix metalloproteinase-9, brain-derived neurotrophic factor, acute ischemic stroke, serum biomarkers

## Abstract

**Background:**

Matrix metalloproteinase-9 (MMP-9) and brain-derived neurotrophic factor (BDNF) have documented roles in the inflammatory injury cascade of neurovascular units following ischemic brain injury. However, their dynamic changes and predictive values after acute ischemic stroke (AIS) have not been well elucidated.

**Objective:**

To investigate the temporal profiles of serum MMP-9 and BDNF concentrations and their relationship with the prognosis in patients with AIS.

**Methods:**

MMP-9 and BDNF levels were measured in 42 AIS patients in prospectively collected blood samples, which were taken on the first day (Day 1), the second day (Day 2), and the fifth day (Day 5) after admission. Healthy subjects (*n* = 40) were used as controls. The AIS patients were divided into groups of good functional prognosis (*n* = 24) and poor prognosis (*n* = 18) according to their modified Rankin Scale score at 3 months. Longitudinal analysis of MMP-9 and BDNF and their association with neurological prognosis was performed using repeated measurement ANOVA.

**Results:**

At baseline (Day 1), the levels of serum MMP-9 and BDNF were significantly higher in the AIS group than in the normal control group (*P* < 0.01). Repeated measurement ANOVA showed a significant main effect and interaction of MMP-9 between good prognosis and the poor group (*P* < 0.05). Further simple-effect analysis showed that the MMP-9 level was significantly increased in the poor prognosis group compared with the good prognosis group at T5 (*P* < 0.05). There were no significant time-dependent or the interaction effect (all *P* > 0.05), but a main effect (*P* < 0.05) for BDNF. Compared with the poor prognosis group, the simple-effect results indicated that the BDNF level of the good prognosis group was lower at Day 1, while the same was reversed for expression at Day 5 (*P* < 0.05).

**Conclusion:**

MMP-9 and BDNF are closely related to the prognosis of patients with AIS in a time-dependent manner. The dynamic changes of the two biomarkers are superior to baseline levels in predicting the prognosis of AIS patients. A sustained decrease in MMP-9 and an increase in BDNF levels in AIS patients after several days of treatment implied a favourable prognosis.

## Introduction

Stroke is still a major public health issue that shortens adults’ lives worldwide (Feigin et al., 2018). The majority of strokes are ischemic, and survivors are more likely to develop permanent disabilities (Diener and Hankey, 2020). Therefore, strategies to accurately risk assess the prognosis of ischemic stroke at the acute stage will be of great value in identifying patients at high risk of poor prognosis promptly and could offer an alternative approach to monitoring patients. Although imaging and conventional cerebrovascular risk factors are helpful in the diagnosis and intervention of ischemic stroke, they cannot fully explain the clinical process and predict outcomes after AIS onset (Guo et al., 2020). Therefore, novel blood biomarkers capable of indicating the likelihood of stroke progression and prognosis have gained interest in recent decades. Multiple interesting markers that are possible to involve neurovascular units (NVUs) impairment cascade after stroke have been identified, such as endothelial dysfunction markers, neural plasticity and vascular remodeling markers and inflammatory markers (Chen et al., 2012; Garcia-Rodriguez et al., 2021; Przykaza, 2021). Among them, inflammatory biomarkers have drawn the most interest, so far none of them have been approved for the clinical management of stroke.

Matrix metalloproteinase-9 (MMP-9) is one of the most extensively studied circulating biomarkers in ischemic stroke because of its role in neuroinflammation, early vasogenic edema, neuronal apoptosis, vascular remodeling, and neurorepair during the ischemic stroke pathobiological process (Vafadari et al., 2016; Montaner et al., 2019; Figiel et al., 2021). Some observations found that higher circulating MMP-9 levels were associated with neurological deficit, infarct volume (Montaner et al., 2001; Morancho et al., 2010), and an increaserisk of poor prognosis (Ning et al., 2006; Guo et al., 2020), while others found no association between MMP-9 and worse outcomes (Tsuruoka et al., 2014; Maestrini et al., 2020). Brain-derived neurotrophic factor (BDNF), a neurotrophin coordinating neuroplasticity in neurological disorders (Farmer et al., 2004; Bramham and Messaoudi, 2005), is another promising blood biomarker that has been investigated in ischemic stroke. Despite the growing evidence for the involvement of BDNF and neuroinflammation in brain disorders (Lima Giacobbo et al., 2019), the association between the circulating level of BDNF and functional outcome after AIS is controversial. Some studies indicated that low serum BDNF levels were associated with large infarct volumes or poor neurological outcomes after stroke onset (Stanne et al., 2016; Qiao et al., 2017; Wang et al., 2017), whereas others did not (Luo et al., 2019; Mourão et al., 2019).

The discrepancy in the findings of the relationship between the circulating MMP-9/BDNF and clinical outcome may partly be due to most studies with a single-occasion measurement of the prognostic metrics, which prevents studying the longitudinal changes of MMP-9 and BDNF during the acute stage of ischemic stroke. The modulations of MMPs and neurotrophin are complex since they may be sensitive to different facets of pathophysiology and may change over time after AIS. Little is known about the temporal behavior of such biomarkers’ expression. The aim of the study was to evaluate dynamic changes in blood MMP-9 and BDNF levels in the acute stage and their association with 3-month prognosis in patients with AIS.

## Materials and methods

### Study population

**FIGURE 1:**
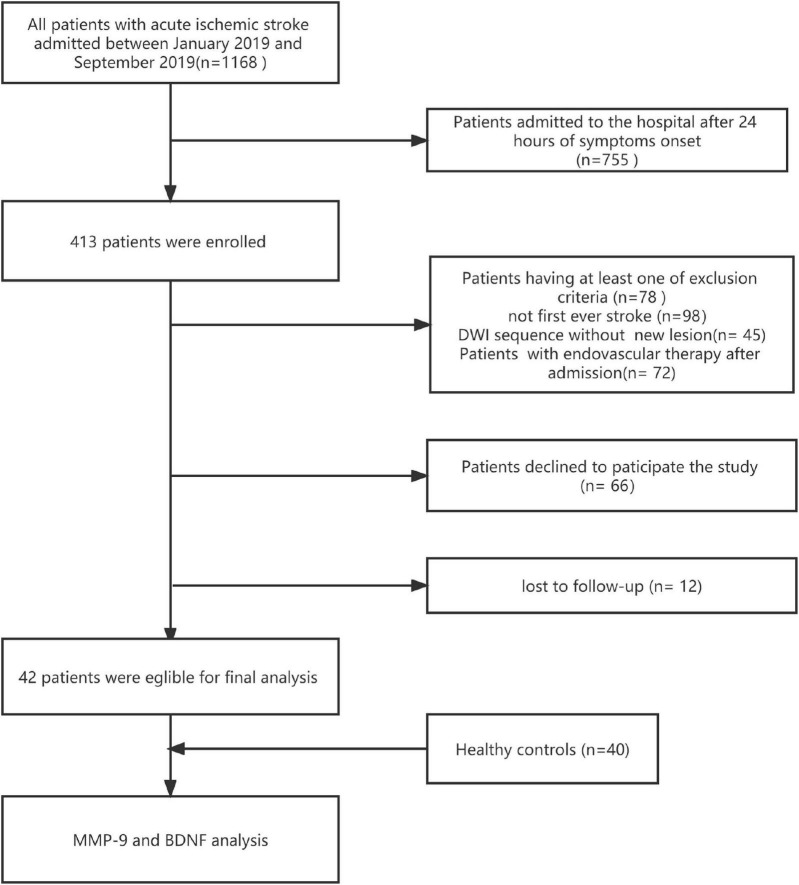
The flow chart of the current study protocol is shown. DWI, diffusion weighted image.

We analyzed data collected in patients with AIS, prospectively recruited within 24 h of symptoms onset between January 2019 and September 2019 in a registered clinical trial (ChiCTR2200055657), a study of biomarkers conducted in the Stroke Center of The First People’s Hospital of Zhaoqing. Ischemic stroke was clinically diagnosed according to the criteria of Trial of Org 10172 in Acute Stroke Treatment, as well as with an acute focal neurological deficit accompanied by neuroimaging evidence from magnetic resonance imaging (MRI) (Adams et al., 1993). The current study’s inclusion criteria were: (a) first-ever ischemic; (b) age over 18 years old; (c) admission to hospital within 24 h of symptoms onset; and (d)completion of at least 3 months of follow-up. Patients with the following conditions were excluded: (a) patients with a history of systemic inflammatory diseases, malignancy, or blood disorders; (b) patients with severe hepatic, renal, or cardiac dysfunction; (c) having received immunosuppressive therapy before admission; (d) with poor treatment compliance; and (e) treatment with endovascular therapy after admission. For comparison, 40 healthy controls without any cardio-/cerebrovascular events, malignant tumors, or autoimmune disease were enrolled during the same period. Figure 1 shows the flowchart of the study participants selection.

The investigation conformed to the Declaration of Helsinki for investigations involving humans. This retrospective study was approved by the Ethics Committee of The First People’s Hospital of Zhaoqing (approval number: B2021-12-01). Each patient and volunteer gave written informed consent.

### Demographic data and clinical assessment

Demographic data of the patients, including age, gender, body mass index (BMI), history of hypertension, history of diabetes mellitus, history of coronary heart disease, smoking, and drinking status, were collected at admission. All blood indices referred to the initial results of the test within 24 h of admission. After admission, patients were treated with urokinase (Rezon Pharmaceuticals, Batch No. 190502) or recombinant human tissue-type plasminogen activator (Boehringer Ingelheim, Batch No. 708765) if eligible for IV thrombolysis administration. All patients received oral aspirin or clopidogrel to prevent thrombosis, and statins to stabilize plaque. Antihypertensive, hypoglycemic, and life guidance were administered according to the patients’ conditions. The modified Rankin Scale (mRS) was applied to assess clinical outcomes in a routine face-to-face visit or standardized telephone interview at 3-months after onset (van Swieten et al., 1988). AIS patients with mRS 0 to 2 at 3-months were considered to have a good prognosis, while those with mRS ≥ 3 were classified into the poor prognosis group in routinely scheduled clinical visits or standardized telephone interviews.

### Blood sampling and marker quantification

Fasting venous blood samples were drawn from patients on the 1st day (Day 1), the 2nd day (Day 2), and the 5th day (Day 5) after admission. Blood samples from healthy controls were taken during a physical examination. After extraction, the samples were allowed to stood for 30 min before being centrifuged at 3,000 r/min for 10 min. The supernatant samples were stored at −80°C until analysis.

Human MMP-9 Enzyme Activity Kit (Shanghai enzyme-linked Biotechnology Co., Ltd., batch No. ml058617m) and human BDNF Enzyme Activity Kit (Wuhan Feien Biotechnology Co., Ltd., batch No. eh0043) were used to determine the MMP-9 and BDNF concentrations in the serum by enzyme-linked immunosorbent assay (ELISA) according to the instructions. The absorbance at 450 nm was measured by a microplate reader, and the concentrations of MMP-9 and BDNF were calculated.

### Statistics

Categorical variables were presented as numbers (percentages), and continuous variables were expressed as mean ± standard deviation (S.D.). A normality test was performed on all continuous variables before analysis. We compared two groups for categorical variables with the chi-square test or Fisher’s exact test when appropriate, and for continuous variables with the *t*-test. The differences in biomarker levels at baseline between AIS patients and the healthy controls were calculated using a *t*-test. MMP-9 and BDNF were normally distributed variables which were assessed by the Shapiro-Wilk test. For repeated measures, group comparisons (good prognosis and poor prognosis) of marker levels at different time points were analyzed by repeated measurement analysis of variance (ANOVA). Simple-effect analysis was also done for comparisons between the good prognosis group and the poor prognosis group at each time point and any of two time points in each group. Data was analyzed using SPSS 22.0 package for Windows (SPSS Inc., Chicago, IL, United States). A *P* value of less than 0.05 (*P* < 0.05) was statistically significant.

## Results

### Demographics and baseline characteristics of the study population

The study population consisted of 42 patients (mean age, 56.81 ± 10.18 years; 20 women [47.6%]) and 40 healthy controls (mean age, 57.15 ± 11.43 years; 20 women [50.0%]). No significant differences in gender and age were found between the AIS patient group and the health control group (*P* > 0.05). Of 42 AIS patients, 18 (53.7%) had a poor prognosis (MRs score at 3 months ≥ 3). The AIS cohort was predominantly male (52.4%),with a history of hypertension (54.7%) at baseline. Baseline demographic and disease characteristics related to the ischemic event did not differ between patients with a good prognosis and those without (Table 1). Of the 42 AIS patients, 12 (29.0%) received IV thrombolysis, and the thrombolysis-treated and -untreated stroke patients were similar in different prognosis groups (Table 1).

**TABLE 1:** Baseline characteristics between good and poor prognosis group.

Indexes	Good prognosis group (n = 24)	Poor prognosis group (n = 18)	*P*
Age (mean ± SD)	56.46 ± 10.03	57.27 ± 10.65	0.802
BMI (mean ± SD)	23.06 ± 3.37	23.34 ± 2.85	0.781
Hypertension (*n*, %)	12(50)	11(61)	0.474
Diabetes mellitus (*n*, %)	5(21)	3(17)	1.000
Coronary heart disease (*n*, %)	0(0)	2(11)	0.178
Smoking history (n, %)			0.531
Non 10 cigarettes/day 20 cigarettes/day >20 cigarettes/day	17(71)	13 (72)	
	1 (4)	1(5.5)	
	5 (21)	3(17)	
	1 (4)	1 (5.5)	
Drinking history (*n*, %)	2 (8)	4 (22)	0.408
SBP, (mean ± SD)	157.96 ± 20.47	148.17 ± 27.54	0.214
DBP, (mean ± SD)	89 ± 14.4	82.94 ± 13.36	0.168
TC, (mean ± SD)	5.02 ± 1.17	5.1 ± 1.16	0.825
HbA1c, median (IQR)	5.6 (5.1, 6.1)	5.9 (5.62, 8.15)	0.118
Thrombolysis, n (%)	7 (29)	5 (28)	0.921

BMI, body mass index; SBP, systolic blood pressure; DBP, diastolic blood pressure; TC, total cholesterol; IQR, interquartile range.

### Time courses of MMP-9 and BDNF and association with clinical outcomes

**FIGURE 2:**
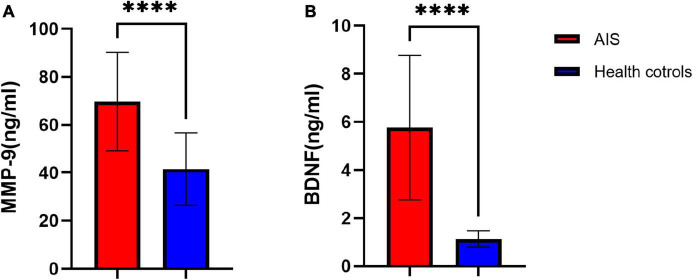
Comparison of baseline serum MMP-9 **(A)** and BDNF **(B)** levels between the AIS group and the healthy control group at baseline. A 42 patients in the AIS group and 40 participants in the health control group were used for the analysis. AIS, acute ischemic stroke; data represent as mean ± SD, ****P < 0.001.

**FIGURE 3:**
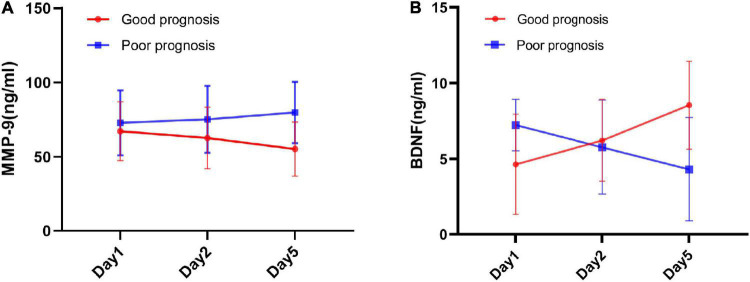
The longitudinal changes of MMP-9 and BDNF over the acute stage of ischemic stroke between good prognosis group and poor prognosis group. **(A)** Mean serum MMP-9 levels changes at Day 1, Day 2, and Day 5 time points between good prognosis group and poor prognosis group. **(B)** Mean serum BDNF levels at Day 1, Day 2, and Day 5 time points between good prognosis group and poor prognosis group. Error bars indicate 95% CI of the mean.

At baseline (Day 1), AIS patients showed higher concentrations of MMP-9 and BDNF than healthy controls (MMP-9 [69.67 ± 20.56 vs. 41.52 ± 15.13], BDNF [5.76 ± 3.00 vs. 1.13 ± 0.35], all *P* < 0.001, Figure 2). Repeated-measures analysis of covariance revealed that, over the acute stage of stroke, serum MMP-9 levels showed substantial increases in the poor prognosis group, which were significantly different from the good prognosis group (*P* = 0.027) (Table 2). We could also see more clearly the trend of MMP-9 levels over time, as shown in Figure 3A. The group × time interaction effect of MMP-9 between the two groups were significant (*P* < 0.05, Table 2), while the time effect was not significant (*P* = 0.053, Table 2). Further simple-effect analysis indicated that, compared with the good prognosis group, the level of MMP-9 in the poor prognosis group showed a significantly increase at the Day 5 time point (*P* < 0.01, Table 2 and Figure 3A). MMP-9 concentrations did not differ between-group at individual Day 1 and Day 2 time points (*P* > 0.05, Table 2).

**TABLE 2:** Difference among Day1, Day2,and Day5 time points for serum MMP-9 and BNDF levels for AIS patients in the good and poor prognosis groups.

	MMP-9 (ng/mL)			BDNF (ng/mL)		
	Day1	Day2	Day5	Day1	Day2	Day5
Good prognosis (*n* = 24)	67.22 ± 19.72	62.75 ± 20.67	55.23 ± 18.11	4.64 ± 3.32	6.22 ± 2.72	8.55 ± 2.90
Poor prognosis (*n* = 18)	72.93 ± 21.80	75.23 ± 22.50	79.82 ± 20.55^ΔΔ^	7.24 ± 1.70^ΔΔ^	5.77 ± 3.11	4.32 ± 3.42^ΔΔ^
Groups effect	*F* = 5.258, *P* = 0.027, η^2^_*P*_ = 0.116			*F* = 0.618, *P* = 0.436, η^2^_*P*_ = 0.015		
Time effect	*F* = 2.236, *P* = 0.113, η^2^_*P*_ = 0.053			*F* = 3.428, *P* = 0.049, η^2^_*P*_ = 0.079		
Interaction	*F* = 31.244, *P* < 0.001, η^2^_*P*_ = 0.439			*F* = 133.954, *P* < 0.001, η^2^_*P*_ = 0.770		

Data represent as mean ± *SD, P*-values denote a significant difference between groups determined by one-way ANOVA, while “^ΔΔ^” denotes a significant difference compared with the good prognosis group under simple-effect analysis (^ΔΔ^*P* ≤ 0.01).

Repeated-measures analysis of covariance revealed that, BDNF levels between the good prognosis and poor prognosis groups were significantly different in the main effect (*P* = 0.015, Table 2). The time effect (*P* = 0.079) and interaction effect (*P* = 0.770) were both non-significant (Table 2). In the acute stage, the BDNF level in the poor prognosis group substantially decreased while it increased in the good prognosis group (Table 2). Figure 3B showed that BDNF levels dropped obviously during the acute stage in the poor prognosis group, which was in marked contrast an good prognosis group. Further simple-effect analysis showed that the BDNF level of the good prognosis group was lower than that of the poor group at the Day 1 time pointand showed an opposite expression at the Day 5 time point (*P* < 0.01, Table 2 and Figure 3B).

### Effect of thrombolysis on serum matrix metalloproteinase-9 and brain-derived neurotrophic factor levels in patients with acute ischemic stroke

**FIGURE 4:**
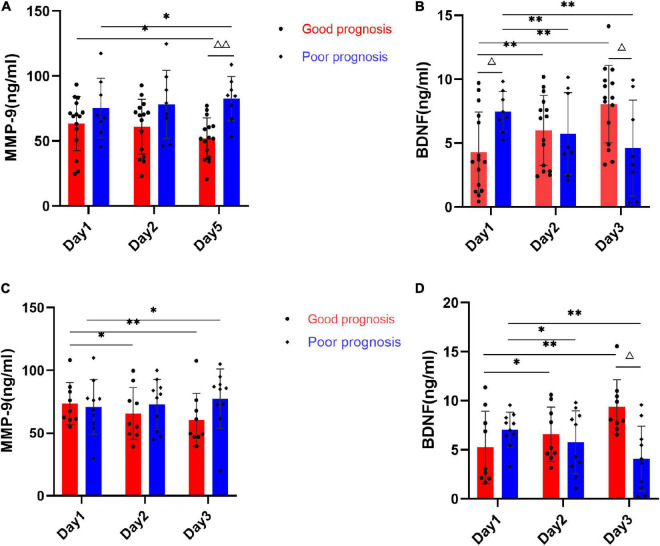
Average serum MMP-9 and BDNF levels of AIS patients with or without thrombolysis at Day 1, Day 2, and Day 5 time points. **(A)** Bar graph shows quantitative analysis for serum MMP-9 of patients with non-thrombolysis between good prognosis group (*n* = 15) and poor prognosis group (*n* = 8). **(B)** Bar graph shows quantitative analysis for serum BDNF of patients with non-thrombolysis between good prognosis group (*n* = 9) and poor prognosis group (*n* = 10). **(C)** Bar graph shows quantitative analysis for serum MMP-9 of patients with thrombolysis between good prognosis group (*n* = 15) and poor prognosis group (*n* = 8). **(D)** Bar graph shows quantitative analysis for serum BDNF of patients with thrombolysis between good prognosis group (*n* = 9) and poor prognosis group (*n* = 10). Data represent as mean ± *SD*, *P < 0.05, ^**^P < 0.01 vs. Day 1 time points, ^Δ^*P* < 0.05, ^ΔΔ^*P* < 0.01 vs. group with good prognosis.

To further indicate that serum MMP-9 and BDNF levels dynamically change in thrombolysis-treated and -untreated stroke patients, subgroup analysis between-group differences at individual time points and within-group changes over time were examined. In the subgroup of patients without thrombolysis, MMP-9 levels showed a significantly increase in patients with poor prognosis compared with patients with good prognosis at the T5 time point (*P* < 0.01, Figure 4A). MMP-9 showed a marginally significant difference from baseline to Day 5 in both prognosis groups (*P* < 0.05). Serum BDNF levels were significantly higher at baseline and conversely lower at Day 5 in the poor prognosis group compared with the good prognosis group (*P* < 0.05, Figure 4B). BDNF showed a substantial decrease in poor prognosis groups (*P* < 0.001), while it increased in good prognosis group over time (*P* < 0.001).

In the subgroup treated with thrombolysis, serum MMP-9 levels were not significantly different between the poor prognosis and good prognosis groups at individual time points (all *P* > 0.05, Figure 4C). MMP-9 showed a decreasing trend in good prognosis and an increasing trend in poor prognosis over time, but not a significant difference (all *P* > 0.05). Moreover, serum BDNF in the poor prognosis group showed significantly lower levels only at T5 time points than in the good prognosis group (*P* < 0.05, Figure 4D). BDNF also showed similar changes as the AIS patients not treated with thrombolysis.

## Discussion

The present study demonstrated that circulating levels of MMP-9 and BDNF were significantly elevated in acute ischemic stroke and has investigated the sequential changes in these two biomarkers after stroke onset and their association with stroke prognosis regardless of whether they are combined with thrombolysis. In the present study, we clarified that elevation of the baseline MMP-9 serum level and its gradual upward trend overtime at the acute stage were associated with a 3-month poor prognosis in AIS patients. BDNF expression levels were found to be higher in the early stages of patients with a poor prognosis compared to those with a good prognosis, but gradually decreased in the later stages. Similarly, this phenomenon was much more significant in the non-thrombolysis group as well.

Over the recent decades, circulating MMP-9 and BDNF concentrations have been found to have potential value in stroke diagnosis and prognosis. However, the prognostic value of circulating expression of MMP-9 in AIS remains elusive. Consistent with our results, a persistent increase in MMP-9 has been reported in stroke (Montaner et al., 2001). Increased peripheral MMP-9 levels have also been reported to be implicated in hemorrhagic transformation after ischemic stroke (Rosell et al., 2008), ischemic stroke severity and infarct volume (Rosell et al., 2005; Demir et al., 2012), and poor prognosis of AIS (Ramos-Fernandez et al., 2011; Gori et al., 2017; Zhong et al., 2017). Nevertheless, the Biostroke study indicated plasma MMP-9 concentrations were not associated with stroke severity and worse outcome (Maestrini et al., 2020). Regarding BDNF as a blood biomarker, inconsistencies are also seen in clinical studies of AIS, which focused on its prognostic impact of adverse outcomes. In the present research, we observed that a substantial decrease in BDNF was involved in the poor outcome at 3 months. For instance, some studies reported that low baseline BDNF was significantly associated with poor functional outcomes at 3 months (Lasek-Bal et al., 2015; Wang et al., 2017), and larger infarct volumes in AIS (Qiao et al., 2017).

At present, researchers have several perspectives on the biological mechanisms underlying the association between these two markers and ischemic stroke. After ischemic brain injury, vascular endothelium impairment leads to release and activation of MMP-9 (Turner and Sharp, 2016). MMP-9 can promote extracellular matrix degradation and increase blood-brain barrier (BBB) permeability, especially early as a mediator of neuroinflammation and later involved in neuroplasticity and vascular remodeling (Lee et al., 2006; Barkho et al., 2008; Guo et al., 2020; Figiel et al., 2021). Experiments in animals have shown that MMP-9 was upregulated in areas of cerebral ischemia and that a breakdown of the blood-brain barrier led to brain swelling, hemorrhage, and neurotoxic cell death in the early stages of stroke (Wang et al., 2020). BDNF may be involved in neuroprotection due to its role in neuronal development, differentiation, and survival and its involvement in homeostatic interactions in neurovascular units (Pikula et al., 2013; Calabrese et al., 2014). Post-ischemic neurogenesis occurs may represent an endogenous repair mechanism for ischemic stroke (Jiang et al., 2016). Experimental studies have demonstrated that administering intravenous BDNF has been shown to facilitate neurogenesis and improve functional recovery after stroke (Schäbitz et al., 2007). In animal models, BDNF levels increased in the brain and circulation immediately after an ischemic incident (Mourão et al., 2019). It was also found that exogenous BDNF affects vasodilation through promoting prostacyclin biosynthesis and protects isolated cerebral artery walls from vasoconstriction-related injury and thrombosis (Santhanam et al., 2010). Accordingly, BDNF may reduce the severity of ischemic stroke through its neurotrophic effects and its vascular protective effects.

The modulation of MMP-9 and BDNF is complex since they may have a pleiotropic and biphasic nature with multiple roles that vary greatly depending on the stage of the AIS. Furthermore, little is known about whether peripheral MMP-9/BDNF variations mirror brain changes in AIS pathophysiology and thus guide clinical interventions. Our findings demonstrated that MMP-9 levels gradually increased from Day 1 to Day 5 in the poor prognosis group, while they gradually decreased in the good prognosis group. There was a significant difference in MMP-9 concentrations between patients with and without a good prognosis on Day 5 after admission, but not at baseline or on Day 2. This finding was consistent with the study of Tsuruoka et al. (2014) which has indicated that increased MMP-9 levels measured at the hyperacute stage (at admission and 24 h after admission) of AIS were not associated with the short-term poor prognosis. Our results suggested that a persistent increased level of circulating MMP-9 could reflect the brain tissue injury and cell death in the acute stage after ischemic stroke (Rempe et al., 2016), which is related to the adverse outcomes rather than baseline expression. Notably, this association might be attenuated by the thrombolytic treatment.

Similar to the previous study (Wang et al., 2017), the current study also demonstrated that a gradually decreasing serum BDNF level after stroke onset appears to be a risk factor for poor prognosis at 3 months. In particular, our study demonstrated the biphasic time course of BDNF changes in patients with different prognoses, with a significant increase in BDNF levels early (Day 1 time point) and a reverse change later (Day 5 time point) in patients with poor prognosis. This association was more significant in patients without thrombolysis. The underlying mechanism of such changes in BDNF expression may be explained as follows: (a) Massive production of circulating BDNF level in patients with poor prognosis in the initial stage may implicate that the excess BDNF found in circulation comes from an alternative, but still speculative, source of peripheral endothelium rather than the brain. Endothelial cells synthesize and release large amounts of BDNF when exposed to oxidative stress and pro-inflammatory cytokines (Béjot et al., 2011a,b). The circulating BDNF at the initial stage after stroke onset may reflect the extent of oxidative stress and pro-inflammatory damage; (b) When the tissue damage is extensive, affecting the affecting the brain’s ability to produce BDNF, circulating decreased BDNF levels may reflect impaired neurogenesis and synaptic plasticity at a later stage of AIS (Calabrese et al., 2014). A previous study also reported that BDNF levels at 72 h of hospitalization but not at admission were associated with hospitalization days in AIS (Mourão et al., 2019). Accordingly, instead of circulating levels of BDNF in the first days after stroke, the sustaining reduction of peripheral BDNF expression at the acute stage is likely to correlate with poor clinical outcomes in patients with AIS.

The strengths of this study lie in the fact that the longitudinal changes of MMP-9 and BDNF are time-sensitive and may be more informative and promising in predicting patients’ prognosis. In addition, our results have indicated that thrombolytic therapy could affect the predictive value of MMP-9/BDNF in AIS, partly due to the fact that thrombolysis therapy may affect the expression of MMP-9 (Saleem et al., 2021). However, the results of our study should be interpreted in light of its limitations. First, the small sample size raises the possibility of undetected bias and does not allow adjustment for all possible confounders, including stroke etiology. In addition, the limited number of patients with thrombolysis may prevent us from examining group differences. Secondly, its single-center, retrospective design may result in selection bias. Although we have investigated the temporal profile of MMP-9 and BDNF concentrations, we emphasize that our results require confirmation in larger sample size and different stroke subtype populations. Furthermore, besides thrombolysis, other pharmacological or non-pharmacological interventions may affect changes in MMP-9 and BDNF. In the current study, we cannot perform an analysis of the impact of such interventions on changes in two biomarkers. Further studies are needed to determine these interventions in the context of their effect on ischemic stroke biomarkers.

In conclusion, MMP-9 and BDNF are closely related to the prognosis of patients with AIS in a time-dependent manner. In the initial stages of AIS, lower serum BNDF levels may predict patients with a favorable prognosis. The decrease in MMP-9 and increase in BDNF levels in the patient after several days of treatment implied a favorable prognosis. In clinical practice, dynamic changes of MMP-9 and BDNF were superior to baseline measurement in predicting the prognosis of ischemic stroke.

## Data availability statement

The raw data supporting the conclusions of this article will be made available by the authors, without undue reservation.

## Ethics statement

The studies involving human participants were reviewed and approved by the Institutional Review Board of The First People’s Hospital of Zhaoqing (B2021-12-01). The patients/participants provided their written informed consent to participate in this study.

## Author contributions

YL and XHa: study concept and design, draft the manuscript, and funding acquisition. SL and HH: acquisition, analysis, and interpretation of the data. XHu and ML: administer the project. YH: supervise the study. YC and ZW: acquisition data and patients’ recruitment. All authors critically revised the manuscript, had full access to all the data in the study, and have read and approved the submitted version.
